# Kinematic analysis of movement patterns during a reach-and-grasp task in stroke patients

**DOI:** 10.3389/fneur.2023.1225425

**Published:** 2023-08-24

**Authors:** Hyoseon Choi, Dongho Park, Dong-Wook Rha, Hyo Suk Nam, Yea Jin Jo, Deog Young Kim

**Affiliations:** ^1^Department of Rehabilitation Medicine, Nowon Eulji Medical Center, Eulji University School of Medicine, Seoul, Republic of Korea; ^2^Department of Rehabilitation Medicine, Yonsei University College of Medicine, Seoul, Republic of Korea; ^3^George W. Woodruff School of Mechanical Engineering, Georgia Institute of Technology, Atlanta, GA, United States; ^4^Institute for Robotics and Intelligent Machines, Georgia Institute of Technology, Atlanta, GA, United States; ^5^Research Institute of Rehabilitation Medicine, Yonsei University College of Medicine, Seoul, Republic of Korea; ^6^Department of Neurology, Yonsei University College of Medicine, Seoul, Republic of Korea

**Keywords:** stroke, upper extremity, biomechanical phenomena, rehabilitation, activities of daily living

## Abstract

**Background:**

This study aimed to evaluate the kinematic movement patterns during a reach-and-grasp task in post-stroke patients according to the upper extremity impairment severity.

**Methods:**

Subacute stroke patients (*n* = 46) and healthy controls (*n* = 20) were enrolled in this study. Spatiotemporal and kinematic data were obtained through 3D motion analysis during the reach-and-grasp task. Stroke patients were grouped using the Fugl-Meyer Assessment (FMA) scale, and a comparison of the groups was performed.

**Results:**

The severe group showed a significantly longer movement time, lower peak velocity, and higher number of movement units than the mild group during the reach-and-grasp task (*p* < 0.05). Characteristic compensatory movement patterns, such as shoulder abduction, thoracic posterior tilting, and upward and external rotation were significantly greater during the forward transporting phase in the severe group than in the mild group (*p* < 0.05). The FMA score was significantly associated with the movement time during the forward transporting phase, number of movement units during the reaching phase, range of shoulder abduction-adduction and wrist flexion-extension movements during the reaching phase, and range of thoracic internal-external rotation during the backward transporting phase (*p* < 0.05).

**Conclusion:**

Post-stroke patients have unique spatiotemporal and kinematic movement patterns during a reach-and grasp-task according to the impairment severity.

## 1. Introduction

Functional impairment after stroke is associated with persistent impairment of the upper extremity. While 40% of post-stroke patients have chronic impairment, only 6% of these patients are satisfied with the functional recovery of their paralyzed upper extremity (1–3). Loss of arm function is associated with the quality of life, as it is essential to perform basic activities of daily living (ADL), such as grasping a cup and bringing it to the mouth.

Motion impairment can be recognized by kinematic assessment, which provides a sensitive, objective, and reliable measurement (4). Although various clinical outcome measures are widely used to estimate the functional impairment of stroke patients, their sensitivity in assessing the degree of motor impairments is lower compared to that of kinematic measurements (5). Kinematic analysis quantitatively enumerates movement control, such as motor performance and movement quality, more accurately through provision of objective data (5, 6). In previous studies, motor performance of hemiparetic patients was found to be slower and less accurate compared to that of healthy subjects, although these patients had only mild impairment when they were clinically assessed with the Medical Research Council Scale or Fugl-Meyer Assessment (FMA) (7, 8). This means that motor abilities can be better identified by motor performance variables than clinical measures. Especially, movement quality measures are useful in identifying sensorimotor controls, including spatiotemporal coordination (9). Therefore, an objective and precise evaluation tool using kinematic analysis techniques is helpful to assess motion impairment as targets for impairment-oriented training in stroke patients.

Recently, kinematic studies on task-based movements of ADL were performed for developing neuroplasticity-based rehabilitation devices, including robots, virtual reality devices, or a brain computer interface. The 3D motion analysis of task-based movements of ADL was investigated (10, 11), with a few studies reporting the kinematic analysis of upper extremities while drinking with a cup in patients with cerebral palsy or minor stroke (12–15). However, studies that performed kinematic analyses in stroke patients with more severe disability have been rarely reported (5, 16).

Thus, the aim of this study was to analyze kinematic differences according to motor impairment severity in hemiplegic stroke patients, including those with severe impairment during the reach-and-grasp task performance.

## 2. Methods

### 2.1. Participants

Post-stroke hemiparesis and healthy controls were recruited from the hospital. The inclusion criteria were (1) patients with first unilateral ischemic or hemorrhagic stroke diagnosed by magnetic resonance imaging or computed tomography scans, (2) patients who were diagnosed 6 months before the study, (3) patients who were able to sit without support, and (4) patients who were able perform the reach-and-grasp task. Exclusion criteria included (1) previous upper extremity surgery, (2) the presence of neurological or musculoskeletal diseases that could affect movement of the upper extremity, and (3) excessive spasticity at any UE joint (Modified Ashworth Scale score > 2).

After assessing the upper extremity function by the FMA for Upper Extremity (FMA-UE, scale 0–66) by one certified occupational therapist, stroke patients were classified into 3 groups according to their FMA-UE scores (mild: 58–66, moderate: 28–57, and severe: 0–27) (17–19). All subjects also underwent the Wolf motor function test (WMFT), Motricity Index (MI), and the trunk control test (TCT) to confirm motor function differences between the groups. All participants signed an informed consent of this cross-sectional study, which was approved by Institutional Review Board (1-2014-0083).

### 2.2. Protocol

**Figure 1:**
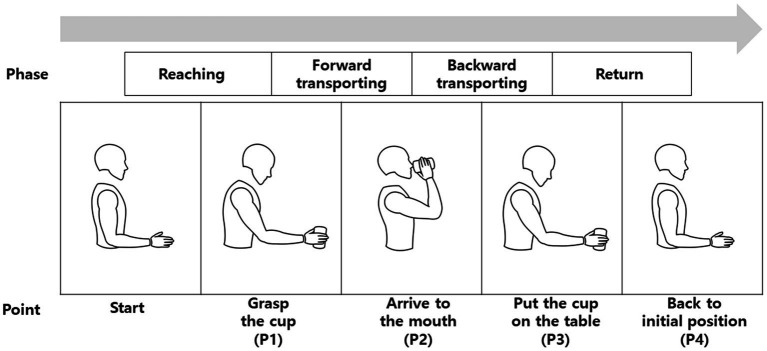
Point and phase definitions of each reach-and-grasp cycle.

Participants were seated on a chair without trunk support. Starting position was with shoulders in a neutral position, elbows at 90° flexion, forearms pronated, and the wrists in a neutral position. A drinking glass (height: 12.5 cm, diameter: 7 cm) was placed 30 cm away along the midline of the body. The reach-and-grasp task comprised the following four phases: reaching, forward transporting, backward transporting, and return; and it included the following four points: when the hand grasps a cup (P1), arrives at the mouth (P2), puts the cup on the table (P3), and returns to the initial position (P4) (Figure 1) (10). Each participant was requested to sit upright and perform the reach-and-grasp task at a comfortable self-paced speed using the hemiparetic arm. The trunk was not restrained, and compensatory movements were allowed if needed. The task was repeated five times.

### 2.3. Data recording and analysis

Eleven reflective markers were placed on the following anatomical landmarks: the spinous process of the seventh cervical vertebra, tenth thoracic vertebra, bilateral clavicular heads, upper sternum, acromion, middle of the humerus, lateral epicondyle, styloid process of the radius and ulna, and the third metacarpophalangeal joint according to the guidelines of the International Society of Biomechanics (20). The 3D marker trajectories were measured by a calibrated six-camera motion analysis system (VICON MX, Oxford Metrics Inc., Oxford, United Kingdom), at a sampling frequency of 100 Hz.

The spatiotemporal and kinematic parameters were determined during the reach-and-grasp task. Spatiotemporal outcome variables included movement time of each phase and movement time of the entire task. The peak velocity for each phase was used to reflect the magnitude of force generated by participants (21). The number of movement units was calculated for all phases using the velocity profiles of the wrist to evaluate the smoothness of movement during each phase. An increased number of movement units indicated a decrease in movement smoothness. The movement unit was determined as a velocity peak where the difference between the previous minimum and the next maximum velocity value was above 20 mm/s in amplitude and the time between two subsequent peaks was longer than 150 ms.The minimum value per phase was 1 unit, and the minimum total was 4 units.

For the kinematic outcomes, we measured the following parameters: angles of the shoulder and wrist joints in the sagittal, coronal, and transverse planes; the elbow joint angle in the sagittal plane, and thoracic angles in tilt, obliquity, and rotation at the four points between each phase; the range of motion (ROM) of the shoulder and wrist joints in the sagittal, coronal, and transverse planes; the elbow joint in the sagittal plane, and the thorax in tilt, obliquity, and rotation during each phase.

We also assessed the sum deviation of each joint angle during the entire task to estimate the degree of movement deviation and compared it with that in healthy participants. Angle deviations were calculated as the difference between joint angles of participants in the patient group and the normal range (defined as mean ± standard deviation for each joint angle) of the 20 healthy participants.

Data were analyzed using MATLAB (MathWorks Inc., Natick, United States). The average of three trials in the middle of five recorded trials was used for statistical calculations. The first trial was only used for familiarization (22). The last trial was not used because of the impact of fatigue (23). The onset/offset movement for each phase was visually identified using frame-by-frame movement inspection by one evaluator (22).

### 2.4. Statistical analysis

A Chi-square test and one-way analysis of variance (ANOVA) were used to evaluate categorical and continuous baseline characteristics. ANOVA with post-hoc analysis using Bonferroni correction was used to compare the spatiotemporal and kinematic parameters among the 4 groups. After the univariate analysis, a multivariate linear regression analysis was performed to identify the spatiotemporal and kinematic parameters significantly associated with the upper extremity impairment level (FMA-UE). Statistical analysis was performed using SPSS, 20.0. A *p*-value <0.05 was considered statistically significant.

## 3. Results

### 3.1. Baseline characteristics

Forty-six participants with post-stroke hemiparesis and 20 healthy controls were enrolled. The mean age of stroke patients and controls was 62.7 ± 13.4 years and 31.5 ± 5.2 years, respectively. There were no significant differences between groups in age, duration after stroke, or lesion type. The FMA, WMFT, and MI scores in the mild group were significantly higher than those in the moderate and severe groups (*p <* 0.001). However, the TCT score was not significantly different between groups. The baseline characteristics of stroke patients are described in Table 1.

**Table 1:** Baseline characteristics of stroke patients.

	Mild (*n* = 16)	Moderate (*n* = 15)	Severe (*n* = 15)	*p*-value
Age (years)	67.44 ± 12.36	61.87 ± 14.28	57.73 ± 13.05	0.134
Man (*n*, %)	12 (75%)	8 (53%)	11 (73%)	0.451
Lesion type, Ischemic (*n*, %)	15 (94%)	13 (87%)	12 (80%)	0.489
Duration (months)	2.19 ± 1.22	2.00 ± 1.13	2.40 ± 1.76	0.737
FMA-UE	61.94 ± 3.02	34.13 ± 5.93^*^	22.00 ± 3.02^*^,^**^	<0.001
WMFT	67.00 ± 9.85	31.13 ± 14.94^*^	19.87 ± 3.23^*^,^**^	<0.001
MI	77.38 ± 7.76	59.20 ± 12.05^*^	41.93 ± 11.00^*,**^	<0.001
TCT	99.19 ± 3.25	92.27 ± 17.35	90.60 ± 20.65	0.271

Values are expressed as mean ± standard deviation. ^*^ Post hoc analysis, compared with the mild group, *p* < 0.05. ^**^ Post hoc analysis, compared with the moderate group, *p* < 0.05. FMA-UE, Fugl-Meyer assessment for upper extremity; WMFT, Wolf motor function test; MI, motricity index; TCT, trunk control test.

### 3.2. Spatiotemporal parameters

#### 3.2.1. Movement time

Stroke patients had longer movement times than healthy participants in all phases and for the whole task (Table 2). The severe group showed significantly longer movement times than the mild group during the reach, forward transporting, and backward transporting phases, and for the entire task (*p <* 0.05); the severe group also showed a significantly longer movement time in the forward transporting phase than the moderate group (*p <* 0.05). Total movement times in the moderate and severe groups were significantly longer than that in the mild group (*p <* 0.05).

**Table 2:** A comparison of spatiotemporal parameters according to the impairment severity.

	Healthy (*n* = 20)	Mild (*n* = 16)	Moderate (*n* = 15)	Severe (*n* = 15)	*p*-value
Movement time (s)					
Reaching	1.00 ± 0.20	1.88 ± 0.54^*^	2.59 ± 1.28^*^	3.07 ± 0.94^*,**^	<0.001
Forward transporting	1.32 ± 0.32	1.55 ± 0.49	2.37 ± 1.03^*,**^	3.52 ± 1.29^*,**,***^	<0.001
Backward transporting	1.28 ± 0.30	1.83 ± 0.47	2.63 ± 1.27^*^	3.20 ± 1.35^*,**^	<0.001
Return	1.13 ± 0.23	1.68 ± 0.59	2.21 ± 1.28^*^	1.95 ± 1.19	0.005
Total	4.74 ± 0.94	6.94 ± 1.71	9.80 ± 4.42^*,**^	11.73 ± 3.02^*,**^	<0.001
Phase ratio (%)					
Reaching	21.28 ± 1.78	27.17 ± 4.62^*^	26.23 ± 4.75^*^	26.54 ± 6.96^*^	0.001
Forward transporting	27.61 ± 2.74	22.16 ± 2.95	24.99 ± 7.09	30.07 ± 9.90^a^	0.004
Backward transporting	27.06 ± 2.55	26.53 ± 3.66	26.84 ± 4.60	26.80 ± 7.00	0.989
Return	24.05 ± 3.16	24.13 ± 5.04	21.94 ± 7.15	16.59 ± 8.19^*,**^	0.002
Peak velocity (mm/s)					
Reaching	626.66 ± 136.82	408.60 ± 116.63^*^	359.19 ± 83.86^*^	340.73 ± 189.13^*^	<0.001
Forward transporting	910.26 ± 149.91	633.00 ± 161.51^*^	483.30 ± 146.65^*^	455.53 ± 204.11^*,**^	<0.001
Backward transporting	851.85 ± 209.21	648.71 ± 226.77^*^	521.58 ± 160.14^*^	524.97 ± 151.80^*^	<0.001
Return	586.16 ± 146.04	537.14 ± 109.34	400.41 ± 154.30^*^	316.34 ± 177.54^*,**^	<0.001
Number of movement units					
Reaching	1.00 ± 0.00	1.19 ± 0.40	2.47 ± 1.25^*,**^	2.73 ± 1.10^*,**^	<0.001
Forward transporting	1.00 ± 0.00	1.31 ± 0.70	2.27 ± 1.71^*^	4.20 ± 1.78^*,**,***^	<0.001
Backward transporting	1.00 ± 0.00	1.31 ± 0.70	2.60 ± 1.68^*^	3.20 ± 1.74^*,**^	<0.001
Return	1.00 ± 0.00	1.25 ± 0.45	1.73 ± 0.88	1.93 ± 1.67^*^	0.017
Total	4.00 ± 0.00	5.00 ± 1.37	9.07 ± 4.18^*,**^	12.07 ± 3.99^*,**,***^	<0.001

Values are expressed as mean ± standard deviation. ^*^ Post hoc analysis, compared with the healthy group, *p* < 0.05. ^**^ Post hoc analysis, compared with the mild group, *p* < 0.05. ^***^ Post hoc analysis, compared with the moderate group, *p* < 0.05.

In healthy participants, the phase ratio during the reaching phase (the relative time spent in each phase) was significantly smaller than that in stroke patients (*p <* 0.05). The phase ratios during the forward transporting and return phases were significantly higher in the severe group than in the mild group (*p <* 0.05, Table 2).

#### 3.2.2. Peak velocity

Stroke patients had significantly lower peak velocities during all phases than healthy participants (*p <* 0.05). Peak velocities in the severe group were significantly lower during the forward transporting and return phases than those in the mild group (*p <* 0.05, Table 2).

#### 3.2.3. Smoothness of movement

Tangential velocity curves were smooth with one predominant peak during every phase in healthy participants. In contrast, stroke patients demonstrated oscillatory velocity curves with multiple peaks. The number of movement units was significantly higher in the severe group than in the mild group during the reach, forward transporting, and backward transporting phases (*p <* 0.001, Table 2); the number of movement units was also significantly higher in the severe group than in the moderate group during the forward transporting phase (*p <* 0.001). The total number of movement units during the entire task was significantly different among the 3 groups, ranging from 4–7, 5–16, and 7–20 in the mild, moderate, and severe groups, respectively (*p* < 0.001, Table 2).

### 3.3. Kinematic parameters

#### 3.3.1. Joint angles and ROMs

**Figure 2:**
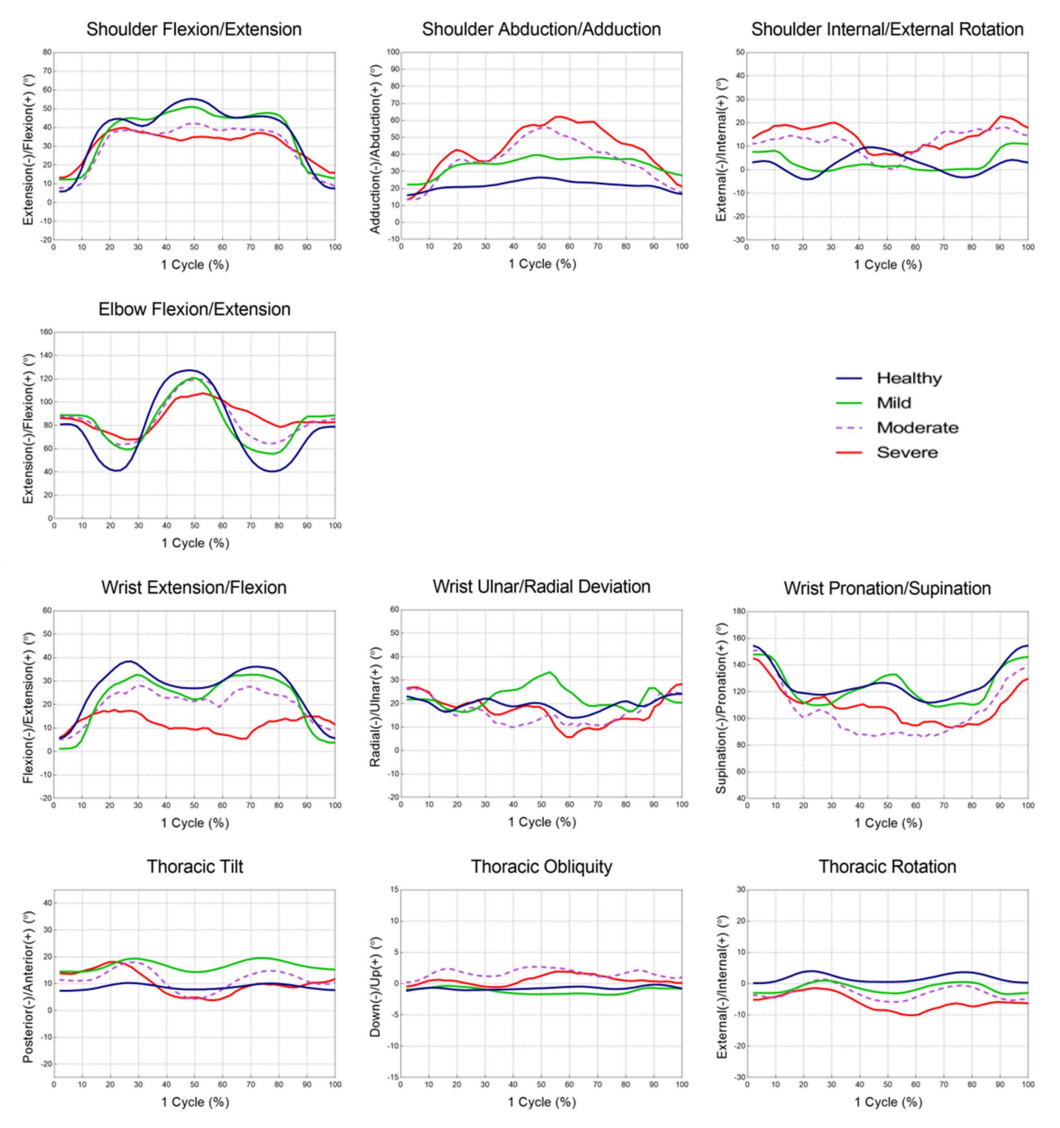
The mean joint angle in upper extremity during the reach-and-grasp task according to the impairment severity.

The joint angles at each point and the ROMs during each phase are shown in Tables 3, 4 and Figure 2. There were relevant differences in the joint angle between each group according to the impairment severity.

**Table 3:** A comparison of joint angles at four points during the reach-and-grasp task according to the impairment severity.

	Healthy (*n* = 20)	Mild (*n* = 16)	Moderate (*n* = 15)	Severe (*n* = 15)	*p*-value
Grasp a cup (P1)					
Shoulder					
Flexion angle	45.37 ± 6.79	40.75 ± 18.08	38.69 ± 10.52	34.71 ± 21.86	0.220
Abduction angle	19.28 ± 10.01	35.83 ± 14.32	38.98 ± 19.35^*^	38.77 ± 28.69^*^	0.005
Internal rotation angle	−0.40 ± 7.92	2.76 ± 16.93	9.15 ± 11.76	21.76 ± 12.46^*,**^	<0.001
Elbow					
Flexion angle	45.30 ± 10.93	65.04 ± 14.41^*^	66.07 ± 15.60^*^	75.15 ± 24.65^*^	<0.001
Wrist					
Extension angle	36.04 ± 10.43	28.39 ± 12.07	25.30 ± 13.74	18.19 ± 11.06^*^	<0.001
Ulnar deviation angle	22.02 ± 11.50	17.91 ± 13.10	15.92 ± 13.42	23.61 ± 13.55	0.313
Pronation angle	118.76 ± 17.49	109.21 ± 24.31	103.17 ± 28.44	120.39 ± 22.73	0.129
Thorax					
Anterior tilt angle	12.93 ± 9.06	19.45 ± 12.08	19.77 ± 9.01	15.69 ± 9.75	0.145
Upward obliquity angle	−1.55 ± 2.63	−0.74 ± 2.81	1.36 ± 3.84	0.27 ± 3.57	0.058
Internal rotation angle	1.87 ± 5.24	1.02 ± 4.92	1.84 ± 5.06	−0.47 ± 4.45	0.511
Arrive at the mouth (P2)					
Shoulder					
Flexion angle	61.05 ± 10.44	46.79 ± 16.96^*^	42.96 ± 7.57^*^	34.64 ± 15.36^*^	<0.001
Abduction angle	41.97 ± 26.41	42.68 ± 18.26	61.25 ± 29.60	75.54 ± 34.53^*,**^	0.002
Internal rotation angle	−8.44 ± 26.73	2.61 ± 15.22	−6.08 ± 14.09	−1.93 ± 21.18	0.415
Elbow					
Flexion angle	125.39 ± 5.62	120.07 ± 10.43	122.79 ± 8.03	111.47 ± 22.73^*^	0.019
Wrist					
Extension angle	25.34 ± 7.42	21.06 ± 10.56	21.22 ± 13.40	8.43 ± 21.00^*,**^	0.005
Ulnar deviation angle	21.36 ± 14.09	29.26 ± 21.29	16.37 ± 23.19	13.14 ± 10.49	0.074
Pronation angle	122.39 ± 18.05	124.14 ± 36.91	91.11 ± 41.30^*,**^	101.79 ± 24.32	0.007
Thorax					
Anterior tilt angle	6.18 ± 5.72	13.83 ± 10.53	5.39 ± 8.65^**^	1.20 ± 9.47^**^	0.001
Upward obliquity angle	−0.68 ± 3.05	−1.15 ± 3.02	3.56 ± 4.36^*,**^	2.61 ± 5.94	0.003
Internal rotation angle	−0.92 ± 4.86	−3.08 ± 5.25	−6.68 ± 6.16	−10.19 ± 10.45^*,**^	0.001
Put the cup on the table (P3)					
Shoulder					
Flexion angle	46.97 ± 6.02	42.62 ± 17.70	39.77 ± 10.52	32.13 ± 19.79^*^	0.027
Abduction angle	21.77 ± 10.19	39.06 ± 14.28^*^	40.39 ± 19.45^*^	40.89 ± 21.35^*^	0.001
Internal rotation angle	0.49 ± 8.95	4.57 ± 17.10	12.90 ± 14.04	19.48 ± 16.96^*,**^	0.001
Elbow					
Flexion angle	42.50 ± 9.88	62.04 ± 13.94^*^	63.19 ± 15.94^*^	75.06 ± 18.82^*^	<0.001
Wrist					
Extension angle	38.15 ± 9.15	30.22 ± 14.03	27.41 ± 15.53	21.23 ± 12.37^*^	0.003
Ulnar deviation angle	22.08 ± 10.39	20.88 ± 13.54	13.89 ± 19.59	18.31 ± 16.86	0.427
Pronation angle	116.25 ± 16.09	111.62 ± 23.27	99.33 ± 33.00	105.91 ± 31.65	0.275
Thorax					
Anterior tilt angle	12.22 ± 7.10	19.40 ± 11.18	18.36 ± 9.58	14.41 ± 15.39	0.188
Upward obliquity angle	−1.69 ± 2.96	−1.12 ± 3.74	2.03 ± 4.93^*^	−1.05 ± 3.86	0.036
Internal rotation angle	2.01 ± 4.92	0.25 ± 4.75	0.45 ± 5.60	−1.21 ± 5.54	0.348
Return to initial position (P4)					
Shoulder					
Flexion angle	8.44 ± 7.92	12.92 ± 12.61	11.55 ± 11.45	15.20 ± 17.88	0.456
Abduction angle	14.09 ± 7.05	28.95 ± 9.39^*^	18.38 ± 8.53^**^	21.03 ± 9.95	<0.001
Internal rotation angle	5.55 ± 7.21	13.49 ± 9.46	13.36 ± 7.75	17.70 ± 11.43^*^	0.002
Elbow					
Flexion angle	78.57 ± 7.16	88.80 ± 7.89^*^	84.75 ± 9.35	81.92 ± 14.36	0.023
Wrist					
Extension angle	6.85 ± 7.21	4.76 ± 8.76	10.47 ± 8.63	13.12 ± 8.04^**^	0.025
Ulnar deviation angle	27.45 ± 4.11	23.03 ± 8.96	23.80 ± 16.99	27.36 ± 12.78	0.551
Pronation angle	153.44 ± 9.19	143.28 ± 12.74	137.51 ± 30.32	128.76 ± 23.86^*^	0.006
Thorax					
Anterior tilt angle	7.85 ± 5.43	14.99 ± 10.02	11.47 ± 8.13	9.37 ± 8.71	0.067
Upward obliquity angle	−1.72 ± 2.97	−0.50 ± 2.97	0.74 ± 2.94	0.32 ± 3.52	0.102
Internal rotation angle	−1.53 ± 4.66	−2.89 ± 4.72	−4.44 ± 3.71	−5.25 ± 4.48	0.076

Values are expressed as mean ± standard deviation. ^*^ Post hoc analysis, compared with the healthy group, *p* < 0.05. ^**^ Post hoc analysis, compared with the mild group, *p* < 0.05.

**Table 4:** A comparison of the range of motion during the reach-and-grasp task according to the impairment severity.

	Healthy (*n* = 20)	Mild (*n* = 16)	Moderate (*n* = 15)	Severe (*n* = 15)	*p*-value
Reaching phase					
Shoulder					
Flexion-extension	39.32 ± 6.61	32.38 ± 11.13	31.93 ± 6.73	29.93 ± 10.68^*^	0.013
Abduction-adduction	8.33 ± 4.11	14.75 ± 8.61	26.73 ± 15.25^*^	34.87 ± 23.11^*,**^	<0.001
Internal-external rotation	7.39 ± 3.99	13.56 ± 8.80	14.53 ± 6.67	20.87 ± 17.06^*^	0.003
Elbow					
Flexion-extension	36.67 ± 13.12	30.19 ± 11.17	26.53 ± 10.40	24.13 ± 16.13^*^	0.032
Wrist					
Extension-flexion	29.46 ± 7.57	29.88 ± 8.59	23.73 ± 12.50	19.27 ± 9.79^*,**^	0.008
Ulnar-radial	12.16 ± 6.07	14.44 ± 9.44	21.07 ± 10.01	17.07 ± 14.78	0.080
Pronation-supination	36.18 ± 14.04	41.44 ± 14.60	56.33 ± 29.69^*^	40.13 ± 24.14	0.044
Thorax					
Anterior–posterior	6.44 ± 7.12	5.38 ± 2.78	8.87 ± 6.61	9.07 ± 6.93	0.276
Upward-downward	1.43 ± 0.78	1.81 ± 1.17	4.53 ± 3.62^*,**^	3.53 ± 1.96^*^	<0.001
Internal-external rotation	3.92 ± 2.11	4.38 ± 2.16	6.60 ± 2.80^*^	6.27 ± 3.08	0.005
Forward transporting phase					
Shoulder					
Flexion-extension	22.68 ± 12.18	12.75 ± 8.20	17.93 ± 10.13	19.33 ± 11.79	0.063
Abduction-adduction	26.58 ± 23.84	15.25 ± 9.30	25.27 ± 18.06	51.73 ± 28.35^*,**,***^	<0.001
Internal-external rotation	26.99 ± 22.48	18.13 ± 9.11	23.53 ± 14.29	34.93 ± 18.44	0.062
Elbow					
Flexion-extension	81.67 ± 12.57	62.44 ± 12.35^*^	58.80 ± 11.42^*^	50.33 ± 17.96^*^	<0.001
Wrist					
Extension-flexion	14.57 ± 5.48	14.69 ± 8.39	21.93 ± 7.38	23.13 ± 15.41	0.076
Ulnar-radial	18.33 ± 11.48	25.19 ± 14.19	30.13 ± 24.28	26.53 ± 14.80	0.742
Pronation-supination	21.54 ± 13.56	38.00 ± 22.64	44.60 ± 32.90	43.20 ± 26.44	0.781
Thorax					
Anterior–posterior	7.71 ± 10.36	5.69 ± 3.40	16.40 ± 10.19^*,**^	17.33 ± 7.80 ^*,**^	<0.001
Upward-downward	1.73 ± 1.94	1.69 ± 0.60	4.67 ± 3.77 ^*,**^	5.60 ± 2.41 ^*,**^	<0.001
Internal-external rotation	3.55 ± 2.39	4.94 ± 2.05	9.20 ± 3.63 ^*^	12.13 ± 8.30 ^*,**^	<0.001
Backward transporting phase					
Shoulder					
Flexion-extension	21.47 ± 13.13	13.44 ± 7.68	15.47 ± 7.68	20.47 ± 11.35	0.084
Abduction-adduction	25.38 ± 22.58	15.25 ± 9.47	24.80 ± 16.29	39.27 ± 23.44^**^	0.009
Internal-external rotation	26.10 ± 21.80	18.13 ± 10.27	26.33 ± 16.76	31.53 ± 17.07	0.201
Elbow					
Flexion-extension	83.92 ± 11.44	63.88 ± 12.02^*^	60.40 ± 13.33^*^	41.27 ± 21.87^*,**, ***^	<0.001
Wrist					
Extension-flexion	14.72 ± 6.66	16.25 ± 9.63	22.60 ± 10.57	19.20 ± 13.78	0.141
Ulnar-radial	16.65 ± 9.57	29.69 ± 18.55	30.53 ± 23.95	22.53 ± 16.40	0.068
Pronation-supination	20.08 ± 11.00	43.44 ± 27.06^*^	46.47 ± 31.38^*^	37.93 ± 26.32	0.008
Thorax					
Anterior–posterior	6.19 ± 8.19	6.62 ± 3.41	14.07 ± 8.55^**^	16.13 ± 12.02^*,**^	0.001
Upward-downward	1.81 ± 1.56	1.56 ± 0.73	4.07 ± 2.96^*,**^	5.60 ± 3.68^*,**^	<0.001
Internal-external rotation	3.78 ± 2.34	4.13 ± 1.86	8.20 ± 2.37	11.80 ± 9.56^*,**^	<0.001
Return phase					
Shoulder					
Flexion-extension	39.18 ± 7.89	33.88 ± 13.31	27.27 ± 11.26^*^	20.47 ± 14.55^*,**^	<0.001
Abduction-adduction	10.16 ± 4.61	13.56 ± 8.13	21.80 ± 13.75	22.60 ± 21.71^*^	0.015
Internal-external rotation	7.99 ± 4.37	16.00 ± 8.76	12.33 ± 8.60	15.00 ± 12.90	0.039
Elbow					
Flexion-extension	37.39 ± 13.00	34.19 ± 14.68	22.27 ± 13.18^*^	14.53 ± 12.79^*,**^	<0.001
Wrist					
Extension-flexion	32.08 ± 7.63	28.31 ± 11.12	21.13 ± 12.73^**^	12.47 ± 8.05^*^,^**^	<0.001
Ulnar-radial	13.05 ± 6.64	19.06 ± 9.48	17.20 ± 16.25	13.93 ± 16.51	0.468
Pronation-supination	39.43 ± 14.93	40.44 ± 16.31	41.13 ± 25.95	28.00 ± 25.22	0.260
Thorax					
Anterior–posterior	5.64 ± 5.65	4.88 ± 2.83	8.07 ± 7.94	9.20 ± 11.29	0.341
Upward-downward	1.80 ± 0.79	2.56 ± 1.59	3.33 ± 4.20	3.53 ± 3.40	0.258
Internal-external rotation	4.28 ± 2.23	4.25 ± 2.70	6.07 ± 3.75	6.13 ± 5.85	0.315

Values are expressed as mean ± standard deviation. ^*^ Post hoc analysis, compared with the healthy group, *p* < 0.05. ^**^ Post hoc analysis, compared with the mild group, *p* < 0.05. ^***^ Post hoc analysis, compared with the moderate group, *p* < 0.05.

During the reaching phase, stroke patients tended to have smaller shoulder flexion, larger abduction, internal rotation motion, and smaller elbow and wrist extension motions than healthy participants. The severe group showed a significantly greater range of shoulder abduction and smaller range of wrist extension motion than the mild group (*p <* 0.05). Stroke patients used more upward and internal rotation motion of the thorax compared to healthy participants. More upward thoracic motion was also observed in the severe group compared to the mild group (*p <* 0.05).

In the forward transporting phase, stroke patients had significantly smaller elbow flexion motion than healthy participants (*p <* 0.05). At P2, stroke patients exhibited smaller shoulder and elbow flexion angles, smaller wrist extension angles, and larger shoulder abduction angles than healthy participants. During the forward transporting phase, the severe group showed a significantly greater range of shoulder abduction than the mild and moderate groups (*p <* 0.001). The severe group also showed significantly larger shoulder abduction and smaller wrist extension angles than the mild group at P2 (*p <* 0.05). The moderate and severe groups showed greater wrist supination angles than the mild group, and the mild and moderate groups showed significant differences between them (*p <* 0.01). Patients in the moderate and severe groups showed greater posterior tilting, and upward and external rotational motion of the thorax than healthy participants and patients in the mild group during the forward transporting phase (*p <* 0.001).

During the backward transporting phase, stroke patients had smaller elbow extension and larger wrist pronation motion than healthy participants (*p <* 0.01). The severe group showed a significantly larger range of shoulder adduction motion and a smaller range of elbow extension motion than the mild group (*p <* 0.01). At P3, larger shoulder internal rotation angles were observed in the severe group than in the mild group (*p <* 0.01). The anterior tilt, downward, and internal rotation ROM values of the thorax were significantly larger in patients in the severe group compared with healthy participants and patients in the mild group (*p <* 0.05).

During the return phase, stroke patients had a smaller range of shoulder extension, elbow flexion, and wrist flexion motion than healthy participants. At P4, stroke patients had larger shoulder internal rotation angles than healthy participants. The severe group showed a smaller range of shoulder extension, elbow flexion, and wrist flexion motion than the mild group (*p <* 0.05).

#### 3.3.2. Angle deviations

The sum of angle deviations from normal data was significantly higher in the severe group than in the mild group for shoulder abduction-adduction and shoulder internal-external rotation (*p <* 0.05, Table 5). The sum of angle deviations in the other joints was not significantly different between groups.

**Table 5:** A comparison of the sum of angle deviations during the reach-and-grasp task according to the impairment severity.

	Mild (*n* = 16)	Moderate (*n* = 15)	Severe (*n* = 15)	*p*-value
Shoulder				
Flexion-extension	267.32 ± 410.06	213.98 ± 168.43	292.88 ± 214.48	0.747
Abduction-adduction	327.40 ± 411.57	380.77 ± 391.51	775.85 ± 691.67^*^	0.041
Internal-external rotation	201.74 ± 181.58	348.91 ± 332.36	532.14 ± 358.31^*^	0.014
Elbow				
Flexion-extension	276.90 ± 135.32	270.93 ± 344.19	436.20 ± 319.61	0.194
Wrist				
Extension-flexion	84.65 ± 95.12	53.30 ± 62.66	91.22 ± 67.43	0.360
Ulnar-radial deviation	209.43 ± 253.23	226.40 ± 323.94	231.32 ± 171.82	0.969
Pronation-supination	551.11 ± 1431.49	435.89 ± 735.83	545.70 ± 498.14	0.940
Thorax				
Anterior–posterior	168.94 ± 340.25	124.37 ± 198.08	194.17 ± 194.63	0.752
Upward-downward	64.38 ± 199.16	32.25 ± 57.75	56.66 ± 72.01	0.773
Internal-external rotation	64.82 ± 121.78	63.30 ± 146.86	123.75 ± 163.86	0.430
Total	2216.69 ± 2364.09	2150.10 ± 1885.56	3188.95 ± 1331.13	0.260

Values are expressed as mean ± standard deviation. ^*^ Post hoc analysis, compared with the mild group, *p* < 0.05.

### 3.4. Spatiotemporal and kinematic parameters associated with impairment severity

The multivariate linear regression analysis showed that the FMA-UE score was significantly associated with the number of movement units, range of shoulder abduction-adduction motion, and wrist flexion-extension motion during the reaching phase. In addition, the movement time during the forward transporting phase and the range of thoracic internal-external rotation motion during the backward transporting phase were also significant parameters associated with the FMA-UE score (adjusted *R*^2^ = 0.802, Table 6).

**Table 6:** Spatiotemporal and kinematic parameters associated with the upper extremity impairment severity (FMA-UE) by multivariate linear regression analysis.

Variables	β (SE)	*p*-value
Adjusted *R*^2^ = 0.802		
Movement time (s) during forward transporting phase	−5.29 (1.16)	<0.01
ROM of shoulder Ab-Ad during reaching phase	−0.36 (0.88)	<0.01
ROM of wrist Fl-Ex during reaching phase	0.50 (0.13)	<0.01
Number of movement units during reaching phase	−4.18 (1.25)	<0.01
ROM of thoracic IR-ER during backward transporting phase	−0.49 (0.20)	0.01

β, regression coefficient; SE, standard error; ROM, range of motion; Ab, Abduction; Ad, adduction; Fl, flexion; Ex, extension; IR, internal rotation; ER, external rotation; FMA-UE, Fugl-Meyer assessment for the upper extremity.

## 4. Discussion

This study revealed kinematic approaches for evaluating the upper extremity motor function in mildly, moderately, and severely impaired stroke patients. Many spatiotemporal and kinematic variables showed significant differences between the respective impairment severities, as well as between stroke patients and healthy controls.

We were able to discriminate the differences in impairment severity in stroke patients with movement times. This finding was consistent with that of previous studies suggesting that movement time can provide information about movement characteristics (8, 12, 13, 24). In a previous study, mild stroke patients showed slower movement times than controls in all phases during the reach-and-grasp task (14). In contrast, the mild group in this study showed no significant differences from healthy subjects except during the reaching phase. In addition, there was no difference in movement time between stroke groups during the return phase, which could be attributed to the smaller ROM in the shoulder and elbow joints or the compensated shoulder internal rotation in the severe group, which boosts the speed during the return phase (22). The phase ratio in the forward transporting phase was greater in the severe group than in the mild group. In contrast, the movement times during the reach and backward transporting phases were not strongly influenced by impairment severity, although the severe group showed slower total movement times.

Peak velocities were lower in the severe group compared to the mild group. A previous study reported that a decreased peak velocity is associated with less torque or force (24). In addition, peak velocity has been shown to increase with remembered movement programs late in motor learning; thus, indicating that it is related to automaticity or programming (16). Our results suggest that impairment may be associated with force generation and programming. Although movement times were significantly different between the mild and severe groups, peak velocities were not significantly different between stroke impairment severities in the reaching and backward transporting phases. These results indicate that other factors affecting movement quality must be considered.

The number of movement units differed according to the impairment severity across all phases except the return phase, which is consistent with results of previous studies (25–28). In Murphy’s study, in which the number of movement units was calculated only for the reaching and forward transporting phases, they found a significant difference between mildly and moderately affected stroke patients, which was correlated with movement times (12). Velocity peaks counted for movement units reflect repetitive acceleration and deceleration during the task and were correlated with movement smoothness and efficiency (12). Previous kinematic studies have demonstrated that smoothness is one of the key measures for quantifying and evaluating movement performance after stroke (15, 29). If each movement is well controlled, the movement time for the task is shorter and movement is smoother (i.e., fewer movement units). Smoothness results from learned inter-joint coordination and increases with motor recovery in stroke patients (30–33). Impairment of joint position sensation is also associated with decreased smoothness of movement (34). In a previous functional imaging study, the activation of secondary motor areas, including ipsilesional premotor cortex, insula and contralesional supplementary motor area, insula, and cerebellum was associated with decreased smoothness during reaching and grasping, indicating that recruitment of additional secondary areas is associated with continuously correcting deviations from optimal movement (35).

In this study, stroke patients had specific movement patterns along with impairment severity of their upper extremities. In the severe group, shoulder abduction with a thoracic upward motion were observed during the reaching phase and these patients exhibited a considerably large shoulder abduction and posteriorly tilted, upward, externally rotated thoracic movement during the forward transporting phase. During the return phase, smaller shoulder extension, elbow flexion, and wrist flexion motions were characteristic movements in the severe group. However, significant thoracic movement to compensate for these motions was not observed during the return phase. These unique movements may have been due to compensation for insufficient shoulder, elbow, and wrist joint motions to complete the task. However, the most important reason for this seems to be poor motor control and the disruption of muscle synergies, crucial aspects of motor impairment in stroke. The alterations in muscle synergies were most prominent in severely impaired stroke patients and less in mild-to-moderately impaired patients in studies of muscle synergy analysis (36).

The shoulder abduction motion to successfully perform the reach-and-grasp task in the severe group was consistent with findings of previous studies (14, 37), which reported larger shoulder abduction in mild stroke patients compared to healthy participants. Further, we found significant deviations of shoulder motions in abduction-adduction and internal-external rotation during the entire task in the severe group compared to the mild group. This information about the sum of angle deviations in stroke patients has not been previously reported. Various mechanisms, such as muscle weakness, loss of selective control caused by altered muscle activation patterns, and/or abnormal muscle tone, may cause differences in shoulder angle deviations between groups (14, 22, 38–41).

In this study, we observed compensatory movement of the thorax in the severe group, which is consistent with findings from previous studies that examined trunk displacement (12, 39, 42, 43). These studies have found that trunk displacement is significantly correlated with stroke impairment severity and that there are differences in trunk displacement between the mild and moderate groups (12, 40). In this study, during the forward transporting phase, we observed posterior tilting, upward, and external rotation of the thorax, which allowed us to differentiate between impairment levels based on this particular movement pattern. While the upper extremity is mainly activated by the contralateral corticospinal pathways, the muscles in the trunk are activated bilaterally to improve the speed, distance, movement quality, and precision. The restraint of excessive trunk movement during training has been demonstrated to enhance arm-trunk control (44, 45). Therefore, it may be important to emphasize thoracic compensatory movements when evaluating movement patterns in stroke patients.

This is the first study to analyze spatiotemporal kinematic parameters of the upper extremity and their association with the impairment severity in stroke patients, including those with severe impairment, using the regression analysis. The number of movement units during the reaching phase may be highly affected by the coordinated movement of the upper extremity. The movement time during the forward transporting phase is significantly affected by the impairment severity because lower muscular strength of patients makes it more difficult to move in the upward direction against gravity. Furthermore, other significant kinematic parameters may be associated with the lack of proximal stability, which leads to ineffective functional movement of the shoulder joint. These variables may provide indirect information on upper extremity impairment levels. Our findings in stroke patients are similar to those of a previous study for children with cerebral palsy, which reported that the total number of movement units and the total time during the reach-and-grasp task were highly correlated with the Manual Ability Classification System score (13, 46). Our findings support the use of the reach-and-grasp task for measuring upper limb impairment levels (47).

The movement characteristics described may elucidate how stroke patients perform impaired motions during ADL. This could suggest directions in selecting kinematic variables for outcome measures in clinical studies and developing a rehabilitation method targeting the impaired motions, including designing upper extremity rehabilitation robotic devices (48–50). For example, customizing and calibrating the device to allow for greater shoulder angle deviation and compensatory movement of the thorax can be beneficial for patients with severe impairment. Moreover, the training program can be conducted by commencing with larger shoulder motions and progressively reducing the deviation over time during the reaching and forward transporting phases within the reach and grasp training program.

### 4.1. Limitations

This study has some limitations. A small sample size and absence of age-matched healthy controls may necessitate further large-scale studies to confirm the findings. Also, kinetic parameters, muscle activation patterns, and differences in temporal measures for each joint were not evaluated. Future studies examining abnormal muscle activation, including co-contraction and temporal measures for each joint during the reach-and-grasp task, are needed. Furthermore, evaluation of movements in various postures or under variable burdens based on purposeful activity is necessary.

## 5. Conclusion

This study suggests that post-stroke hemiplegic patients have unique spatiotemporal and kinematic movement patterns with compensation when performing the reach-and-grasp task, which can be used to plan individual treatment programs and evaluate treatment effects during rehabilitation.

## Data availability statement

The raw data supporting the conclusions of this article will be made available by the authors, without undue reservation.

## Ethics statement

The studies involving humans were approved by Severance hospital human research protection center. The studies were conducted in accordance with the local legislation and institutional requirements. Written informed consent for participation was not required from the participants or the participants’ legal guardians/next of kin in accordance with the national legislation and institutional requirements.

## Author contributions

HC was responsible for the study conception, design, and wrote the original article. YJ collected clinical data of this study. HC and DP were responsible for data analysis. D-WR, HN, and DK participated in experimental design and provided advice during the experiment procedure. DK did final approval of the manuscript. All authors contributed to the article and approved the submitted version.

## Conflict of interest

The authors declare that the research was conducted in the absence of any commercial or financial relationships that could be construed as a potential conflict of interest.

## Publisher’s note

All claims expressed in this article are solely those of the authors and do not necessarily represent those of their affiliated organizations, or those of the publisher, the editors and the reviewers. Any product that may be evaluated in this article, or claim that may be made by its manufacturer, is not guaranteed or endorsed by the publisher.
